# Validation of a Proteomic-Based Prognostic Model for Breast Cancer and Immunological Analysis

**DOI:** 10.1155/2023/1738750

**Published:** 2023-12-16

**Authors:** Yunlin Yu, Linhuan Dong, Changjun Dong, Xianlin Zhang

**Affiliations:** Department of General Surgery, Affiliated Renhe Hospital of China Three Gorges University, Yichang 443000, China

## Abstract

Breast cancer (BC) has emerged as an extremely destructive malignancy, causing significant harm to female patients and society at large. Proteomic research holds great promise for early diagnosis and treatment of diseases, and the integration of proteomics with genomics can offer valuable assistance in the early diagnosis, treatment, and improved prognosis of BC patients. In this study, we downloaded breast cancer protein expression data from The Cancer Genome Atlas (TCGA) and combined proteomics with genomics to construct a proteomic-based prognostic model for BC. This model consists of nine proteins (HEREGULIN, IDO, PEA15, MERIT40_pS29, CIITA, AKT2, CD171 DVL3, and CABL9). The accuracy of the model in predicting the survival prognosis of BC patients was further validated through risk curve analysis, survival curve analysis, and independent prognostic analysis. We further confirmed the impact of differential expression of these nine key proteins on overall survival in BC patients, and the differential expression of the key proteins and their encoding genes was validated using immunohistochemical staining. Enrichment analysis revealed functional associations primarily related to PPAR signaling pathway, steroid hormone metabolism, chemokine signaling pathway, DNA conformation changes, immunoglobulin production, and immunoglobulin complex in the high- and low-risk groups. Immune infiltration analysis revealed differential expression of immune cells between the high- and low-risk groups, providing a theoretical basis for subsequent immunotherapy. The model constructed in this study can predict the survival of BC patients, and the identified key proteins may serve as biomarkers to aid in the early diagnosis of BC. Enrichment analysis and immune infiltration analysis provide a necessary theoretical basis for further exploration of the molecular mechanisms and subsequent immunotherapy.

## 1. Introduction

Breast cancer (BC) is one of the most common malignant tumors worldwide [1]. It has been reported that the incidence of breast cancer is increasing at a rate of 0.5% per year, with approximately 2 million people diagnosed with breast cancer each year [2]. Breast cancer poses a particularly serious threat to women and is one of the leading causes of cancer-related death among females [3]. Although the incidence of BC is higher in developed countries compared to developing countries, the mortality rate is significantly lower. Early detection and timely treatment can prevent 28% to 37% of breast cancer deaths in these countries [4]. Currently, mammography and breast ultrasound play important roles in early screening for breast cancer [5]. However, they have some notable limitations, such as being subjective to the interpreting physician's bias and being costly [6, 7]. Therefore, it is crucial to search for more accurate, objective, and cost-effective diagnostic and prognostic biomarkers to aid in the early detection and treatment of breast cancer.

Abnormal protein expression plays a crucial role in biological processes such as metabolism, immune response, and biological signal transduction, making it one of the most prominent factors in the occurrence and development of diseases [8]. The study of proteomics is aimed at elucidating the interactions between different proteins and their roles within an organism, providing us with more detailed information to deepen our understanding of the nature of diseases [9]. Furthermore, proteomic research holds significant potential in various applications, including early disease diagnosis, personalized treatment, and dose determination [10, 11]. This field offers promising prospects for advancing the field of medicine. Protein expression can be inferred by studying mRNA expression, but there are significant differences between mRNA expression levels and protein expression levels [12]. With advancements in proteomic technologies, we can gain a more comprehensive and in-depth understanding of the protein landscape within organisms. This helps address the issues of data inaccuracy and insufficient abundance often encountered in traditional mRNA transcriptome studies due to factors such as RNA stability and post-transcriptional modifications [12]. Therefore, exploring more accurate biomarkers through proteomic research in breast cancer holds significant importance.

Bioinformatics is a field that harnesses advanced computational techniques to mine and analyze extensive datasets generated from various “omics” platforms, including genomics, transcriptomics, proteomics, and metabolomics [13]. Its primary aim is to identify novel diagnostic and prognostic biomarkers. The rapid evolution of bioinformatics has empowered us to distinguish between different subtypes of breast cancer, such as basal-like and luminal B breast tumors, indicating potential variations in patient prognoses [14]. Furthermore, we have successfully identified protein products located within noncoding genomic regions, thereby offering robust support for the discovery of novel tumor-specific immunotherapeutic targets. By amalgamating multidimensional data, bioinformatics can unveil concealed patterns, associations, and biological features that may elude detection through traditional laboratory methodologies [15, 16]. These encompass changes in RNA expression levels and alterations in DNA methylation status [17]. This comprehensive approach holds the promise of identifying biomarkers that could fundamentally transform disease diagnosis, prognosis, and treatment selection. The application of bioinformatics provides a fresh perspective to the field of medicine, paving the way for advancements in personalized medicine and precision therapy.

In this study, a bioinformatic analysis was performed to identify nine differentially expressed prognostic proteins. A breast cancer survival prognostic model was constructed, and the effectiveness and accuracy of the model were evaluated. Immunohistochemical staining confirmed the differential expression of the encoding genes of the key proteins between normal breast tissue and breast cancer tissue. Subsequently, the breast cancer immune microenvironment and immunotherapy were explored. Our research provides evidence and future directions for early diagnosis, disease prognosis, immunotherapy, and potential molecular interactions in breast cancer.

## 2. Materials and Methods

### 2.1. Acquisition and Processing of Microarray Data

To acquire the transcriptome and proteome datasets of breast cancer, as well as the corresponding clinical information, we accessed The Cancer Genome Atlas (https://portal.gdc.cancer.gov/). The dataset included 112 samples of normal tissue and 1100 samples of breast cancer tissue.

In our study, the inclusion criteria for data selection entailed the incorporation of samples exclusively sourced from individuals who had received a pathological diagnosis of breast cancer. We accorded priority to samples providing comprehensive and complete clinical information, encompassing patient demographics, histopathological data, and clinical attributes. Additionally, the samples included in our analysis were required to possess corresponding sequencing and proteomic data, including gene expression profiles and protein expression profiles, which were deemed indispensable components for our comprehensive analytical approach. Conversely, our exclusion criteria were systematically implemented to ensure the precision and integrity of our data. Samples with missing or incomplete clinical or molecular data were rigorously excluded from our study. Furthermore, our exclusion criteria encompassed patients with co-occurring primary medical conditions, a history of prior treatments, or any factors that might introduce interference or bias into our analysis. All data utilized in this study were sourced from TCGA database. To enhance the depth and diversity of our dataset and to facilitate cross-validation, we randomly allocated samples into two groups: the train group and the test group. This approach ensured the quality, comprehensiveness, and statistical power of our research.

Using the Perl software (https://www.perl.org/), we extracted the clinical information of the samples and organized the protein expression data. Next, we utilized the “Limma” and “impute” packages in R language to merge the protein expression data with the survival data in the breast cancer dataset. The “Limma” package provides functions for differential expression analysis, while the “impute” package offers methods for missing data imputation. By integrating the protein expression data and survival data, we aimed to investigate the potential association between protein expression profiles and patient survival outcomes in breast cancer. This analysis would allow us to identify potential prognostic markers and provide insights into the underlying molecular mechanisms of breast cancer progression and prognosis.

### 2.2. Selection of Key Proteins and Construction of Prognostic Model

After preprocessing the data, we conducted the single-factor Cox proportional hazard analysis and Kaplan-Meier survival analysis on the preprocessed dataset. These analyses are aimed at identifying proteins that showed significant associations with patient survival outcomes. The protein candidates exhibiting significant associations were determined based on their *p* values. Furthermore, the expression levels of these proteins were recorded. To determine the optimal number of features (proteins) for the prognostic model, we employed Lasso regression, a regularization method that selects the most relevant features while shrinking the coefficients of less informative features.

### 2.3. Validation of Prognostic Model Accuracy

Survival analysis was performed on the samples using R programming language with the “survival,” “survminer,” and “timeROC” packages. Risk curves, including risk scores, survival status plots, and risk heatmaps, were generated using the “pheatmap” package. Independent prognostic analysis was conducted using the “survival” package. Univariate Cox proportional hazards regression and multivariate Cox proportional hazards regression models were employed to perform independent prognostic analysis based on age and tumor stage. Receiver operating characteristic (ROC) curve analysis was performed using the “survival,” “survminer,” and “timeROC” packages. Finally, column line plots were generated for clinical data and risk files after data organization using the “regplot” and “rms” packages in R programming language.

### 2.4. GSEA

The data were cleaned and prepared using the gene symbol file, risk file, and gene set files (including GO.symbols and KEGG.symbols). GSEA functional and pathway enrichment analyses were performed on these files using R packages such as “limma,” “org.Hs.eg.db,” “clusterProfiler,” and “enrichplot.”

### 2.5. Survival Analysis of Prognostic Key Proteins and Identification of Encoding Genes

The samples were divided into high-risk and low-risk groups based on the expression levels of the target proteins, and KM survival analysis was performed for each protein in the model. The encoding genes of the nine key proteins, which were used to construct the prognostic model, were standardized using the Gene Expression Omnibus (GEO) database (https://www.ncbi.nlm.nih.gov/).

### 2.6. Immunohistochemical Staining Was Performed to Validate the Differential Expression of IDO1 (IDO) and NRG1 (HEREGULIN)

We selected 10 clinical pathology diagnosed breast cancer patients from the Department of General Surgery, Renhe Hospital, Three Gorges University, for the period of 2019-2022. Inclusion criteria for breast cancer patient selection are consisted of the following: histopathological confirmation of excised tissue (we exclusively enrolled breast cancer patients with a histopathological confirmation to ensure the accuracy of disease classification) and breast tumor stage and subtype (patients representing various breast cancer stages and subtypes were chosen to reflect the clinical heterogeneity of the disease). This encompassed both early-stage (I and II) and late-stage (III and IV) breast cancer, as well as patients with different molecular subtypes, such as ductal carcinoma, HER2-positive breast cancer, and triple-negative breast cancer. Exclusion criteria included incomplete clinical information (patients with missing or incomplete clinical data, including tumor staging, treatment history, and follow-up information, were systematically excluded to ensure comprehensive dataset integrity) and concurrent medical conditions (patients with concurrent medical conditions that could potentially significantly affect the interpretation of immunohistochemical staining results were excluded). Paraffin blocks of breast cancer samples were obtained, and the corresponding adjacent noncancerous tissues were selected as normal controls for intragroup comparison. The rabbit anti-IDO1 antibody (catalog number 13268-1-AP) and rabbit anti-NRG1 antibody (catalog number 10527-1-AP) were purchased from Wuhan Sanying Biotechnology Co., Ltd.

### 2.7. Analysis of Immune Cell Infiltration

In the immune cell infiltration analysis, we performed 1000 simulations of immune cell infiltration using the “e1071” and “preprocessCore” packages. The resulting data on risk assessment and immune cell infiltration for all samples were then organized using the “ggpubr” package. Subsequently, the “ggpubr” package was employed to conduct differential analysis of immune cell infiltration.

### 2.8. Immunotherapy Analysis and Sample Typing Analysis

We downloaded the immune scoring files from The Cancer Immunome Atlas (TCIA) (https://tcia.at/home) and performed analysis and visualization using the “ggpubr” package. To differentiate the subtypes of the samples, we used the “limma” and “ConsensusClusterPlus” packages and classified them based on the expression levels of the model proteins. We conducted survival analysis using the subtype classification result file and plotted survival curves.

## 3. Result

### 3.1. Screening Prognostic Proteins and Constructing Prognostic Models

**Figure 1:**
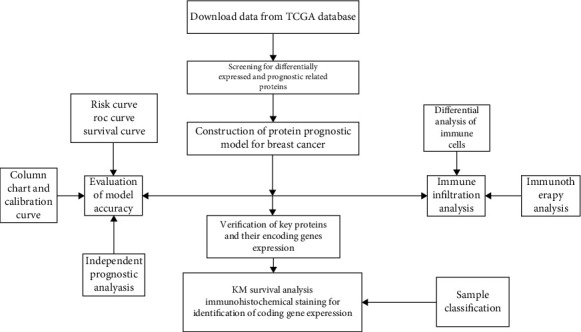
Flow chart of the design and evaluation of this study.

The transcriptomic and proteomic datasets of breast cancer, along with the corresponding clinical information, were extracted, processed, and analyzed from TCGA database.  Figure 1 shows the research flow chart.

**Figure 2:**
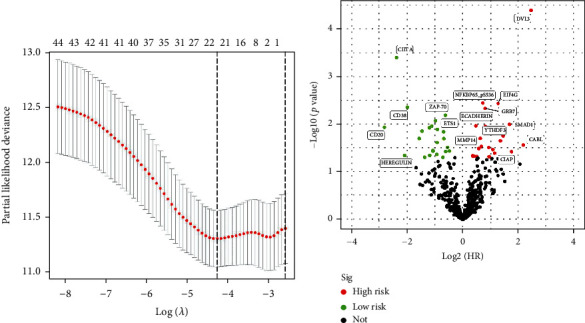
Construction of prognostic risk model for breast cancer: (a) Lasso regression model shows the number of target proteins; (b) significantly different proteins in the volcanic map.

Nine proteins related to breast cancer prognosis were identified through the single-factor Cox regression analysis and Kaplan-Meier survival analysis. In the figure, red color indicates high-risk proteins (HR > 1), green color indicates low-risk proteins (HR < 1), and proteins with significant differential expression (*p* < 0.05) are labeled with their names. All significantly differentially expressed proteins are presented in the volcano plot of Figure 2(b) (with log2(HR) on the *x*-axis and −log10(*p* value) on the *y*-axis). For example, NFKBP65_pS536 is a high-risk protein, and higher expression levels indicate greater risk for patients. On the other hand, HEREGULIN is a low-risk protein, and higher expression in breast cancer suggests a lower risk of disease for patients. The Lasso regression model reveals the point with the minimum cross-validation error, which represents the number of selected feature proteins, as shown in Figure 2(a). The multivariate Cox regression analysis demonstrates the involvement of HEREGULIN, IDO, PEA15, MERIT40_pS29, CIITA, AKT2, CD171, DVL3, and CABL in constructing the prognostic model (Supplementary Table 1) The model's risk score is calculated as follows: HEREGULIN^∗^ (−1.450) + CABL^∗^ (1.636) + IDO^∗^ (−0.326) + PEA15^∗^ (−0.895) + MERIT40_pS29^∗^ (−0.833) + CIITA^∗^ (−1.599) + Akt2^∗^ (−0.551) + CD171^∗^ (−0.309) + DVL3^∗^ (0.781).

### 3.2. Survival Analysis between Groups

**Figure 3:**
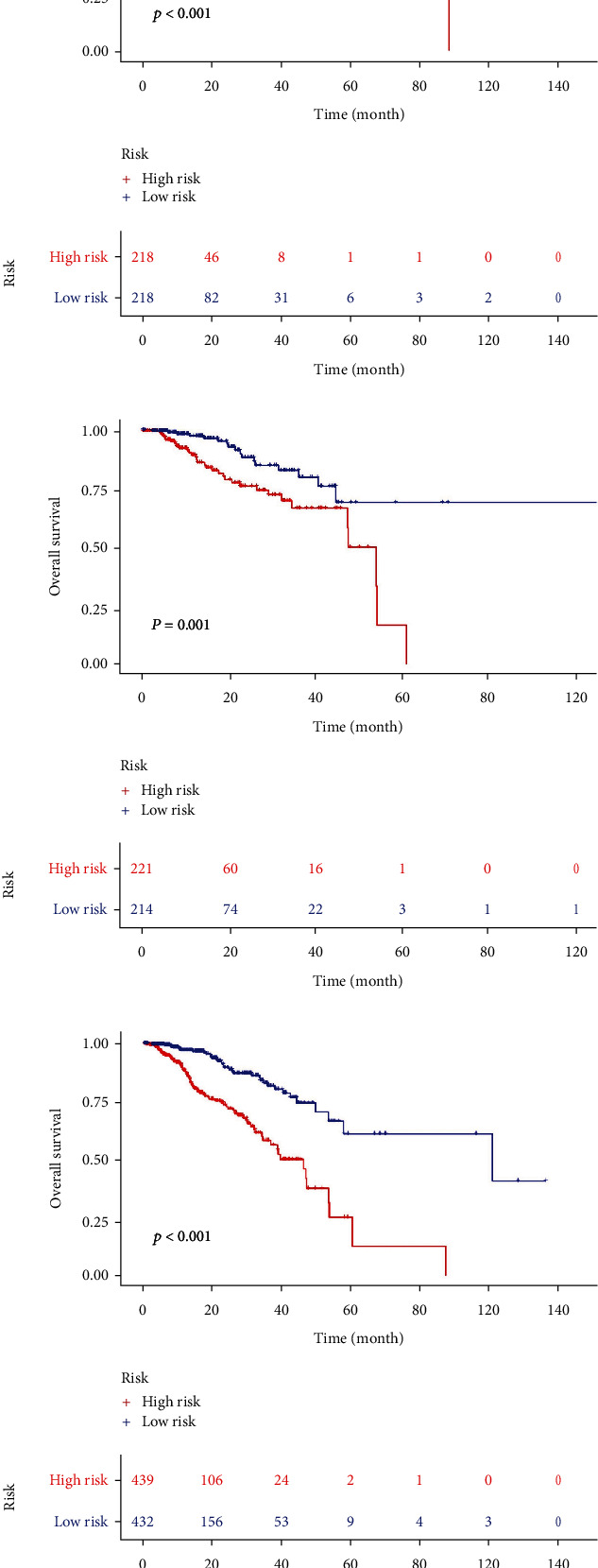
Survival analysis of the train group, test group, and total sample group: (a) survival analysis chart for the train group; (b) survival analysis chart for the test group; (c) survival analysis chart for total samples.

The train group, test group, and overall sample group were stratified into high-risk and low-risk groups based on the median risk score (Figures 3(a)–3(c)). The Kaplan-Meier survival analysis was performed on these three groups, and the results are shown in the figure. The survival time difference between the high-risk and low-risk groups is statistically significant, with *p* values < 0.05 (red color represents the high-risk group, and blue color represents the low-risk group, with survival time on the *x*-axis and survival rate on the *y*-axis). These findings indicate that the constructed prognostic model can accurately distinguish patients between the high-risk and low-risk groups. Compared to the low-risk group, the high-risk group exhibits a significant decrease in survival rate.

### 3.3. Building a Prognostic Risk Model

**Figure 4:**
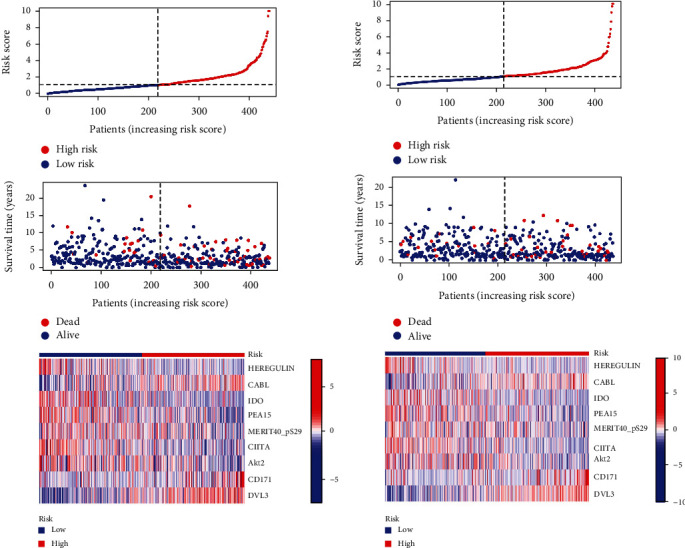
Construction of prognostic risk model for breast cancer. (a) Train group: distribution of risk scores and heatmaps of 9 proteins based on the survival status of patients with risk scores and the construction of prognosis models. (b) Test group: distribution of risk scores and heatmaps of 9 proteins based on the survival status of patients with risk scores and the construction of prognosis models.

The accuracy of the prognostic model was further evaluated by plotting risk curves, survival status graphs, and risk heatmaps. As shown in Figures 4(a) and 4(b), an increase in the risk score is associated with a poorer survival status and an increased number of patient deaths. The risk heatmap reveals that DVL3, CD171, and CABL are high-risk proteins, while HEREGLUIN, IDO, PEA15, MERIT40_pS29, CIITA, and AKT2 are low-risk proteins. Higher expression of the low-risk proteins is associated with a lower risk for patients. The results from the train group and test group confirm the further refinement of the evaluation dimensions of the prognostic risk model.

### 3.4. Univariate and Multivariate Cox Regression Analyses

**Figure 5:**
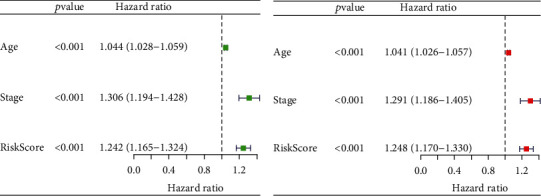
Evaluation of prognostic risk model for breast cancer. (a) Univariate regression analysis to evaluate prognosis models. (b) Multivariate regression analysis to evaluate prognosis models.

To evaluate whether the risk prognostic model is independent of other clinical characteristics as independent prognostic factors, we performed single-factor Cox regression analysis on the risk file. The results, as shown in Figure 5(a), indicate that clinical features such as age, tumor stage, and risk score are associated with survival time and survival status. The multivariate Cox regression analysis in Figure 5(b) further confirms that age, tumor stage, and risk score (all with *p* values < 0.05) can serve as independent prognostic factors (Supplementary Table 2). This also suggests that the constructed model can function as an independent prognostic factor, separate from other clinical characteristics.

### 3.5. Receiver Operating Characteristic Analysis

**Figure 6:**
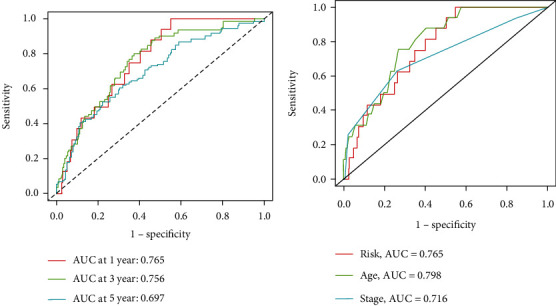
Accuracy evaluation of prognostic risk model for breast cancer: (a) time receiver operating characteristic; (b) risk score receiver operating characteristic.

The ROC curve (area under the curve) can assess the accuracy of our constructed model in predicting patient survival. As shown in Figure 6(a), the area under the ROC curve for 1-year, 3-year, and 5-year survival is 0.765, 0.756, and 0.697, respectively. In Figure 6(b), the area under the ROC curve for risk score, age, and tumor stage is 0.765, 0.798, and 0.716, respectively. These results confirm the effectiveness of the prognostic model in prediction.

### 3.6. Risk Model Column Chart and Calibration Curve

**Figure 7:**
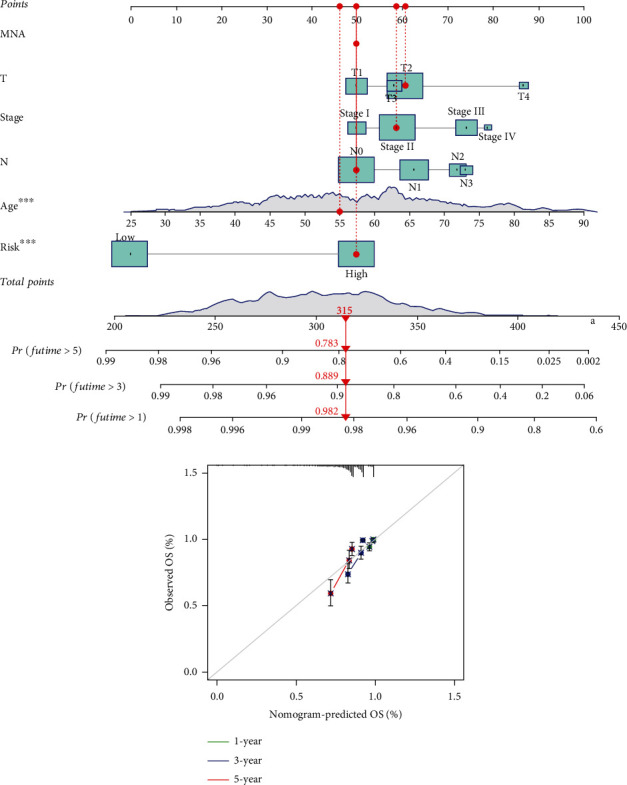
Evaluation of the effectiveness of prognostic risk model for breast cancer: (a) risk model column chart; (b) calibration curve.

The column diagram (alignment diagram) integrates multiple indicators and accurately predicts patient survival time. As shown in Figure 7(a), the scores for each clinical feature and the risk score are obtained based on individual scoring scales, which further predicts the survival rates of patients at 1 year, 3 years, and 5 years. The calibration curve is a method to evaluate the accuracy of the column diagram prediction. In Figure 7(b), the calibration curves for 1 year, 3 years, and 5 years are close to the reference line, indicating a high accuracy of the column diagram in predicting patient survival time. These overall results indicate that the risk prognostic model has a high accuracy in predicting the prognosis and survival time of breast cancer patients.

### 3.7. Survival Analysis of 9 Key Proteins

**Figure 8:**
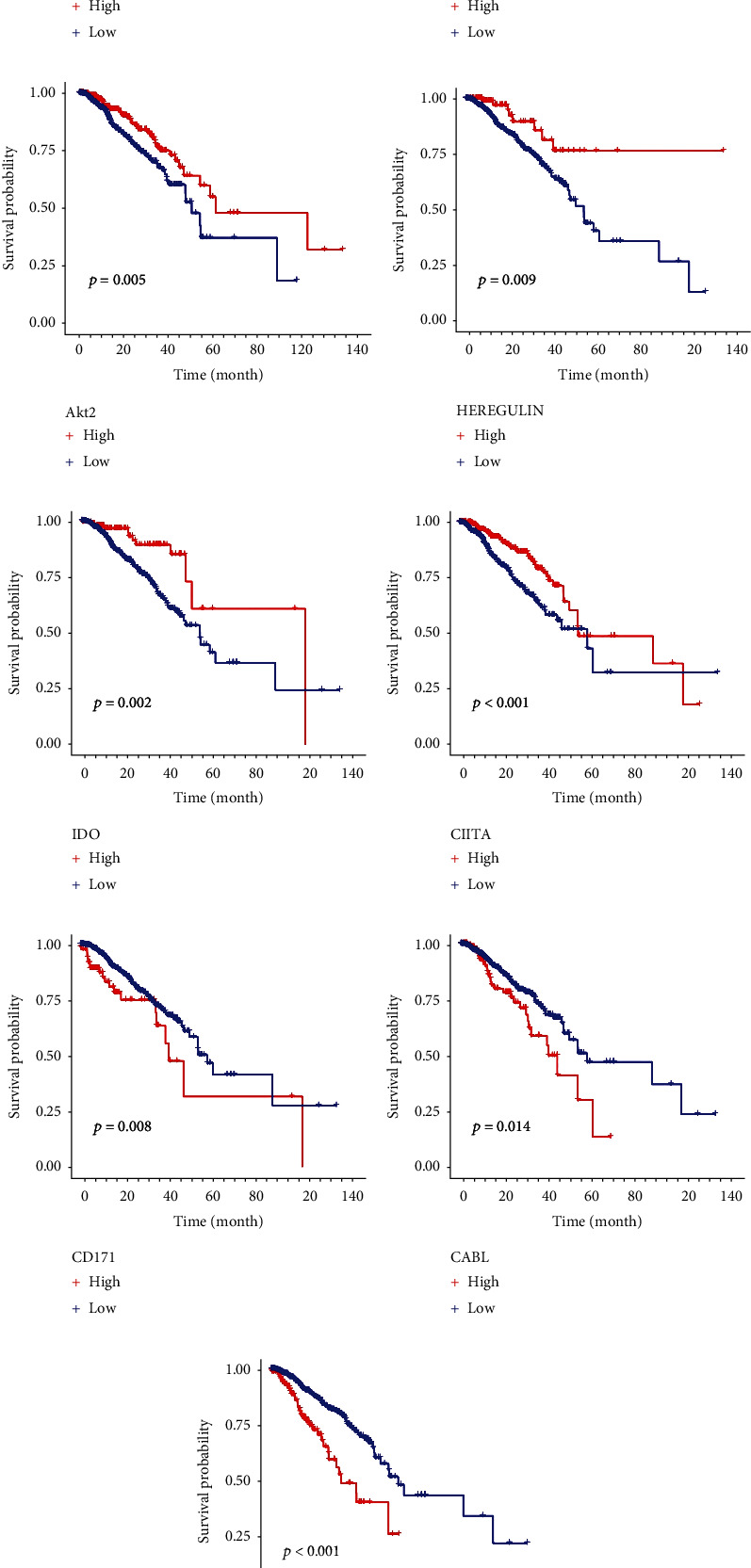
Relationship between the expression of nine proteins and survival analysis in breast cancer: (a–h) survival analysis of nine key prognostic proteins KM.

In order to clarify the relationship between the expression of key proteins in the prognostic model and overall survival, the samples were divided into high-expression group and low-expression group based on the median expression level of the key proteins. As shown in Figures 8(a)–8(h), the KM survival curves for the 9 key proteins are presented. Among them, high expression of HEREGLUIN, IDO, PEA15, MERIT40_pS29, CIITA, and AKT2 is associated with higher overall survival rates, while high expression of DVL3, CD171, and CABL is associated with lower survival rates. Furthermore, the *p* values for these associations are all less than 0.05, indicating that the differential expression of these proteins is significantly correlated with the overall survival of breast cancer patients.

### 3.8. Key Protein Coexpression Analysis

**Figure 9:**
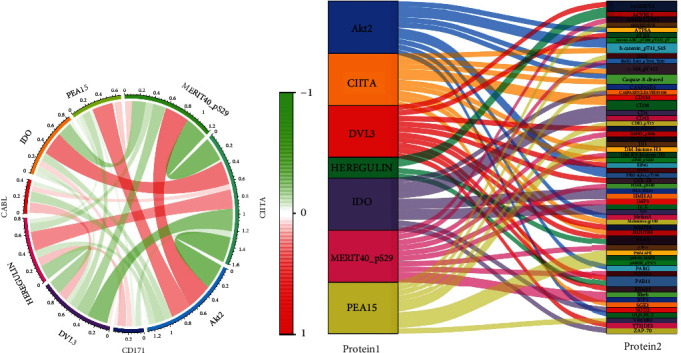
Graph of the relationship between 9 key proteins and other proteins: (a) coexpression circle diagram of key proteins; (b) sangi diagram of key protein coexpression with other proteins.

In order to further understand the interaction between these 9 proteins, the coexpression analysis results are shown in Figure 9.  Figure 9(a) displays the correlation between the 9 key proteins, while Figure 9(b) further illustrates their interactions with other proteins, leading to their biological effects. The encoding genes of the 9 prognostic-related proteins were standardized using the high-throughput gene expression database GEO (https://www.ncbi.nlm.nih.gov/geo/): NRG1 (HEREGULIN), IDO1 (IDO), PEA15 (PEA15), MERIT40_pS29 (MERIT40), CIITA (CIITA), AKT2 (AKT2), DVL3 (DVL3), L1CAM (CD171), and ABL1 (ABL1).

### 3.9. KEGG Enrichment Analysis

**Figure 10:**
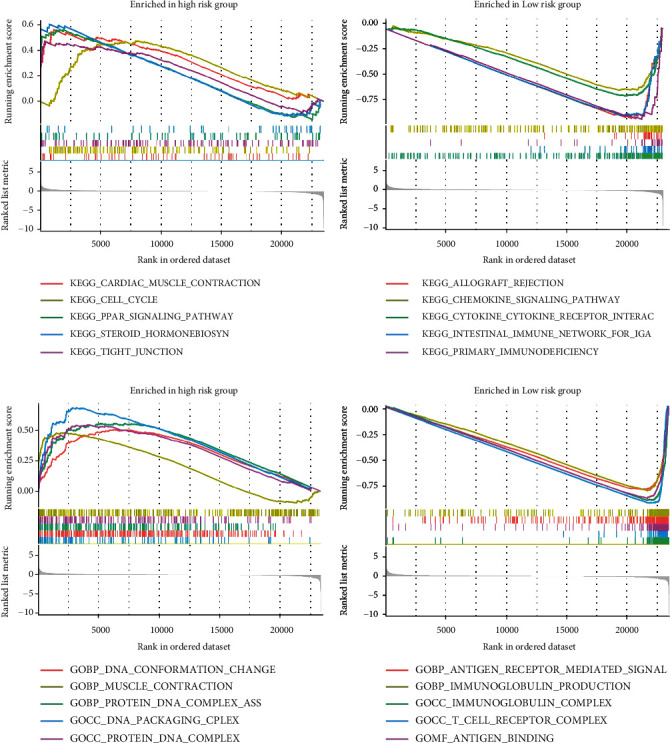
Enrichment analysis of high- and low-risk groups: (a) analysis of pathway enrichment in high- and low-risk groups; (b) analysis of functional enrichment in high- and low-risk groups.

The KEGG pathway enrichment analysis, as shown in Figure 10(a), reveals the pathways closely associated with the high-risk group, including the PPAR signaling pathway, cell cycle pathway, cardiac contraction, and steroid hormone synthesis. In contrast, the low-risk group exhibits activity in pathways such as intestinal immune network for IgA production, chemokine signaling pathway, graft-versus-host disease, and cytokine-cytokine receptor interaction. In Figure 10(b), the GO functional enrichment analysis demonstrates that in the high-risk group, these genes are mainly involved in processes such as protein and DNA complex formation, DNA conformational changes, muscle contraction, and protein-DNA complex interactions. In the low-risk group, these genes are closely associated with biological behaviors such as antigen receptor-mediated signaling pathway, immunoglobulin production, immune complex formation, and T cell receptor complex.

### 3.10. Immunohistochemical Staining Analysis of the Expression of Two Differential Proteins

**Figure 11:**
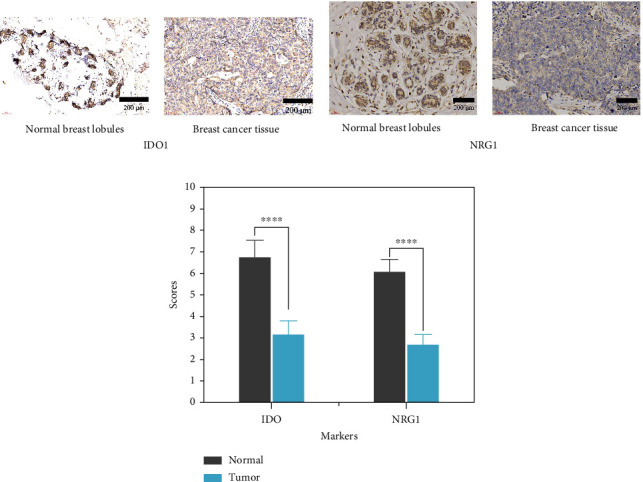
Immunohistochemical analysis of two differential protein expressions: (a) immunohistochemical staining detection of IDO1 expression in normal breast tissue and breast cancer tissue; (b) immunohistochemical staining detection of HRG1 expression in normal breast tissue and breast cancer tissue.

Based on the GEO database, HEREGLUIN (NRG1) and IDO (IDO1) were found to have the most significant differential protein expression. Ten diagnosed BC samples from Renhe Hospital affiliated to Three Gorges University were selected for immunohistochemical staining using paraffin-embedded tissue sections. Tumor tissues and their corresponding adjacent normal tissues were used as normal controls. The results in Figure 11(a) demonstrate that IDO1 (IDO) protein exhibits strong positive staining in breast normal tissues and shows significant differential expression compared to the corresponding cancer tissues. The results in Figure 11(b) show that NRG1 (HEREGULIN) protein exhibits strong positive staining in breast normal tissues. The results were visualized as a bar graph based on grayscale scores, as shown in Figure 11(c).

### 3.11. Differential Expression of Immune Cells in BC

**Figure 12:**
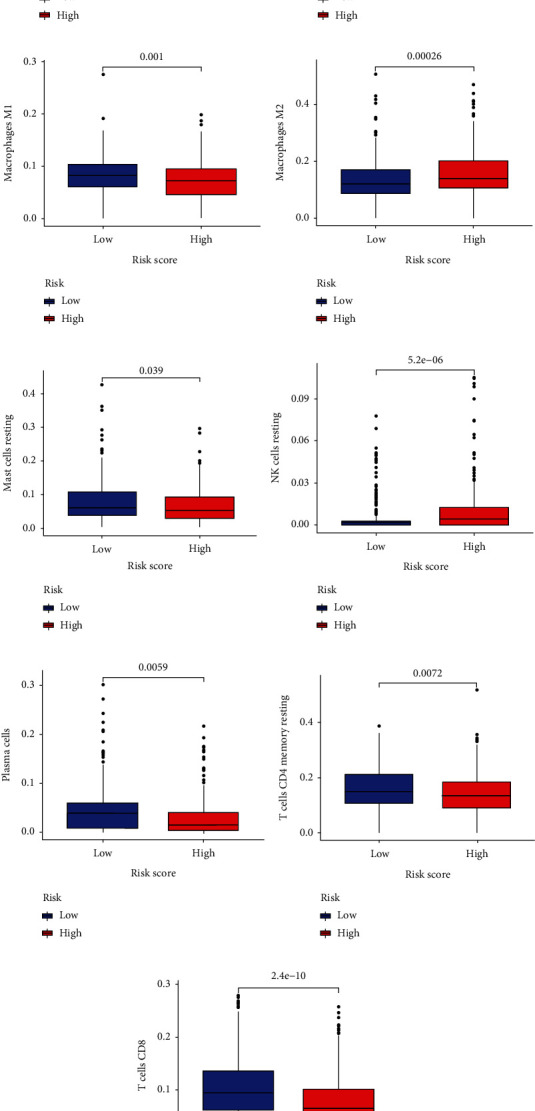
Differential expression of different immune cells in breast cancer: (a–i) differential analysis of immune cells.

Based on the analysis of immune cell differences (as shown in Figures 12(a)–12(i)), we found differential expression of immune cells between the high-risk and low-risk groups. Immune cell types such as immature B cells, dendritic cells, macrophages, M1 macrophages, M2 macrophages, mast cells, NK cells, plasma cells, CD4^+^ cells, T cells, CD8^+^ T cells, and T follicular helper cells exhibited differential expression.

### 3.12. Results of Immunotherapy Analysis

**Figure 13:**
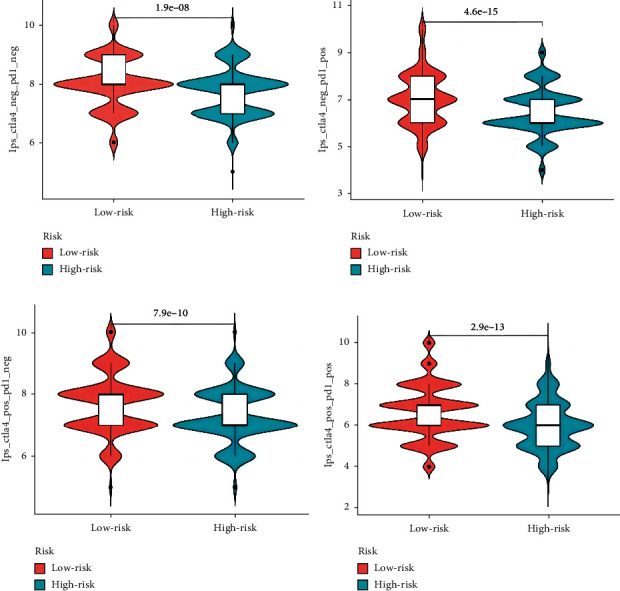
Differences in immunophenotype scores between low-risk and high-risk groups. (a–d) Immunotherapy analysis violin chart.

The results of immune therapy analysis using The Cancer Immunome Atlas (TCIA) (https://tcia.at/home) data are shown in the figure. In Figure 13(a), both CTLA4 immune checkpoint and PD1 are negative, indicating that patients in this group are not sensitive to these two immune therapies. In Figure 13(b), CTLA4 is negative and PD1 is positive, suggesting that patients in this low-risk group respond well to PD1-targeted therapy. In Figure 13(c), CTLA4 is positive and PD1 is negative, indicating that patients in this low-risk group respond better to CTLA4-targeted therapy. In Figure 13(d), both CTLA4 and PD1 are positive, suggesting that patients in this low-risk group have the best response to combined CTLA4 and PD1 therapy.

### 3.13. Sample Classification

**Figure 14:**
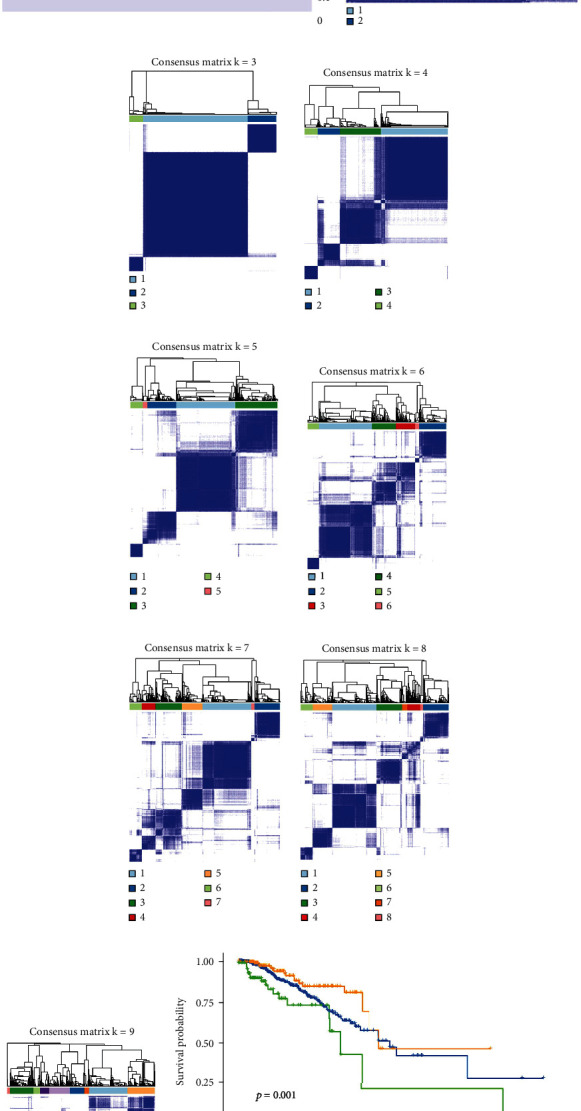
Evaluation of sample typing effectiveness: (a–i) sample typing analysis; (j) sample KM survival analysis after typing.

The sample subtyping results (shown in Figures 14(a)–14(i)) demonstrate that when we categorize the samples into three different subtypes, this classification provides the greatest benefit for subsequent treatments. Based on this sample subtyping, we conducted KM survival analysis (as shown in Figure 14(j)). The results indicate a significant difference in survival time among the different subtypes (*p* < 0.05).

## 4. Discussion

This study employed protein expression data, transcriptomics, and clinical data from breast cancer patients in TCGA database to conduct an analysis aimed at investigating the pathogenesis and prognosis of breast cancer, as well as identifying potential candidate proteins for tumor screening. The findings of this study have the potential to offer new options for the early diagnosis and timely treatment of BC.

We performed KM survival analysis and univariate COX analysis to screen for 9 prognostic-related proteins. Furthermore, we utilized Lasso regression and multivariable COX analysis to establish a prognostic risk model for BC. The accuracy of the prognostic model was further evaluated using risk scores, survival status plots, and risk heatmaps. To assess whether the risk prognostic model was independent of other clinical features as an independent prognostic factor, we conducted univariate COX regression analysis. The accuracy of predicting patient survival was further evaluated using ROC curves.

Various validation results demonstrated that the prognostic model constructed in this study serves as an independent prognostic factor and effectively evaluates patient survival. KEGG pathway enrichment analysis revealed that the PPAR signaling pathway was enriched in the high-risk group, focusing on regulating gene expression related to energy homeostasis, lipid metabolism, and inflammation, providing insights into molecular interactions and targets within this pathway [18]. The active chemokine signaling pathway was observed in the low-risk group, providing information on the regulation of receptors, intracellular signaling molecules, and cellular processes related to inflammation, immune cell recruitment, and tissue development [19].

Functional enrichment analysis indicated that DNA conformational changes were highly active in the high-risk group, involving processes such as DNA bending, twisting, or unwinding, which may occur during DNA replication, transcription, or DNA-protein interactions. In the low-risk group, activation of immunoglobulin function was prominent, primarily contributing to the synthesis and production of immunoglobulins (antibodies) in biological processes [20].

Through immune infiltration and immune therapy analysis, we discovered differences in immune cell expression between the high-risk and low-risk groups defined by our model. It is noteworthy that CD8^+^ T cells play a crucial role in the immune system and work together with T follicular helper cells to mediate cellular and humoral immune responses [21]. The differential expression of immune cells between the high-risk and low-risk groups may reflect distinct immune reactions and pathological processes. For example, in certain autoimmune diseases, macrophages and T cells may play a critical role as they participate in the regulation of inflammatory and autoimmune responses. The differential expression of immune cells between the high-risk and low-risk groups may be associated with different immune regulatory mechanisms. For instance, CD4^+^ T cells and dendritic cells are key players in immune regulation as they can recognize and modulate the function of other immune cells [22].

Studies have shown that the prognostic proteins identified in this study are involved in the progression of malignant tumors. NRG1, a neuroregulatory protein, serves as the major physiological ligand for human epidermal growth factor receptor 3 (HER3). It can induce dimerization of ErbB2/HER2 and ErbB3/HER3 receptors, leading to their constitutive activation and subsequent modulation of downstream signaling pathways such as phosphoinositide 3-kinase-protein kinase B (PI3K-AKT) and mitogen-activated protein kinase (MAPK), thereby influencing cellular processes including growth, proliferation, apoptosis, migration, and angiogenesis [23–25].

Indoleamine 2,3-dioxygenase 1 (IDO1) is a rate-limiting enzyme containing cytosolic heme, and its main function is to catalyze the degradation of tryptophan to kynurenine [26]. Kynurenine is typically involved in cell signaling pathways as a neurotransmitter and molecule in the first step of tryptophan degradation [27]. Studies analyzing cancer genomics have found that high expression of IDO1 indicates poor prognosis in colorectal cancer, but in certain hormone-related cancers such as breast cancer and ovarian cancer, higher IDO1 expression is associated with significantly prolonged survival and better prognosis compared to lower IDO1 expression levels. This may suggest a relationship between IDO1 and hormone expression, but research in this area is limited both domestically and internationally, indicating a potential direction for future investigation [28].

Proline-rich protein 15 (PEA15), enriched in astrocytes, is a small protein expressed widely in mammals and has been shown to affect the localization of ERK1/2 [29]. By promoting the accumulation of activated ERK1/2 in the cytoplasm, PEA-15 can inhibit tumor cell invasion and proliferation [30], and it has been demonstrated to suppress the occurrence of triple-negative breast cancer [31]. Additionally, PEA15 can induce autophagy in human ovarian cancer cells, thereby extending patients' overall survival [32].

Class II transactivator (CIITA) is a transacting factor that participates in the transcriptional activation of major histocompatibility complex class II (MHC-II) genes by binding to specific transcription factors [33]. In previous studies, the inactivation of CIITA played a crucial role in lymphomas originating from thymic medullary B cells. These tumors often exhibit genomic alterations, including structural genomic rearrangements, missense, nonsense, and frameshift mutations in 53% of primary tumor biopsies and PMBCL-derived cell lines [34].

Akt, also known as protein kinase B (PKB), belongs to the AGC family of protein kinases. Akt consists of three isoforms: Akt1 (PKB*α*), Akt2 (PKB*β*), and Akt3 (PKB*γ*) [35]. It acts downstream of phosphoinositide 3-kinase (PI3K) and regulates various cellular processes, including cell proliferation, cell survival, metabolism, tumor growth, and metastasis. The PI3K/Akt2 signaling pathway is frequently dysregulated in breast cancer and plays a significant role in breast cancer development and progression [36, 37].

Disheveled (DVL) proteins are highly conserved and typically divided into three isoforms: DVL1, DVL2, and DVL3. They are considered central intracellular effectors of the Wnt signaling pathway [38]. Studies have shown that human mutations in DVL1 primarily affect craniofacial development, while DVL3 mutations can cause short stature. In mice, DVL1 is mainly expressed in the neuroectoderm [39].

L1 cell adhesion molecule (L1CAM) is a neural cell adhesion molecule, also known as a cell recognition molecule, belonging to the immunoglobulin superfamily of integral membrane proteins with characteristic adhesive and signaling properties [40]. It plays a crucial role in cell migration, proliferation, and differentiation during early stages of neural system formation [41].

ABL1 is primarily expressed in the nucleus and cytoplasm and is involved in cell differentiation, stress response, and other processes. Research has indicated that the SH3 domain of the ABL1 protein negatively regulates its function, and high expression of ABL1 often indicates poor prognosis, which is commonly observed in chronic myeloid leukemia, but it is less studied in other cancers [42].

In the treatment process of breast cancer, chemotherapy regimens should be personalized based on the patient's cancer type, stage, overall health, and individual needs. Selection of appropriate chemotherapy drugs and treatment courses depends on factors such as the subtype of breast cancer (e.g., hormone receptor-positive and HER2-positive) and the stage of the disease (early-stage or advanced). Combining multiple chemotherapy drugs is often employed to enhance treatment efficacy and reduce the risk of drug resistance [43, 44]. Commonly used chemotherapy agents include paclitaxel, cyclophosphamide, doxorubicin, and docetaxel [45]. Postoperative treatment for breast cancer is a comprehensive process. Providing psychological support, nutritional counseling, and rehabilitation services on the medical front can aid patients in coping with the challenges of the treatment process and improving their overall quality of life [46].

The study confirms that the regulation of specific miRNAs might be influenced by healthy diet and physical exercise, closely correlating with the survival rates of patients [47]. Additionally, the upregulation and downregulation of certain circRNAs are associated with adverse prognosis in breast cancer, indicating their potential as biomarkers for chemotherapy resistance [48]. Furthermore, lncRNAs play an indispensable role in the development of breast cancer. The extensive variations in protein expression levels within breast cancer cells constitute a primary driving force behind their malignant transformation [49]. Alterations in RNA expression levels drive changes in various cellular processes, including abnormal protein synthesis and degradation, signaling pathways, metabolism, DNA repair, and apoptosis [50]. In breast cancer research, the significance of transcriptomic, proteomic, and system biology studies cannot be overstated, as they are crucial for gaining in-depth insights into the intricate molecular mechanisms underlying protein imbalance in breast cancer.

In future research, we intend to delve deeper into the correlation between dysregulated proteins identified in our study and the determinants of treatment failure. This will encompass their potential involvement in mechanisms of drug resistance and treatment response. Based on existing literature reports, the prognostic proteins identified in our study play roles in various diseases, and our research further explores their expression and functions in BC.

## 5. Conclusion

In this study, we employed bioinformatic methods to screen for prognostic-related proteins in BC and developed a prognostic risk model. We validated the model's ability to predict prognosis and risk in BC patients. Subsequently, we conducted a series of expression analyses on these feature proteins, which provide novel insights for early diagnosis and treatment of BC patients. Enrichment analysis and immune infiltration analysis helped broaden our understanding of the molecular mechanisms underlying BC and identify potential therapeutic targets in clinical practice. In future studies, we plan to conduct animal in vivo experiments and examine clinical specimens to explore a new approach for the early diagnosis and treatment of BC.

## Data Availability

The datasets presented in this study can be found in online repositories. The names of the repository/repositories and accession number(s) can be found in the article.

## Supplementary Materials

Supplementary 1 Table 1: multivariate COX analysis to obtain prognostic model proteins.

Supplementary 2 Table 2: multivariate COX regression analysis results of age, tumor staging, and risk score.
