# Supplementary material for: Validation of a Proteomic-Based Prognostic Model for Breast Cancer and Immunological Analysis
Source: Int J Genomics. 2023 Dec 16;2023:1738750. doi: 10.1155/2023/1738750 (PMC10748720; doi:10.1155/2023/1738750)
Supplement: Supplementary 1 — Table 1: multivariate COX analysis to obtain prognostic model proteins. [file 1738750.f1.docx]

| **id** | **coef** |
| --- | --- |
| HEREGULIN | -1.45000423239843 |
| CABL | 1.63645355830813 |
| IDO | -0.326203889892655 |
| PEA15 | -0.895017234299396 |
| MERIT40_pS29 | -0.833524646396537 |
| CIITA | -1.59927552449126 |
| Akt2 | -0.551718610757205 |
| CD171 | 0.309991889375248 |
| DVL3 | 0.781316140238698 |
