# Supplementary material for: Validation of a Proteomic-Based Prognostic Model for Breast Cancer and Immunological Analysis
Source: Int J Genomics. 2023 Dec 16;2023:1738750. doi: 10.1155/2023/1738750 (PMC10748720; doi:10.1155/2023/1738750)
Supplement: Supplementary 2 — Table 2: multivariate COX regression analysis results of age, tumor staging, and risk score. [file 1738750.f2.docx]

| **id** | HR | HR.95L | HR.95H | pvalue |
| --- | --- | --- | --- | --- |
| **Age** | 1.04109173869677 | 1.02578888359099 | 1.05662288383194 | 9.81692967317467e-08 |
| **Stage** | 1.29059992871179 | 1.29059992871179 | 1.40469602882908 | 3.58600678857063e-09 |
| **riskScore** | 1.24763871091051 | 1.17006661106336 | 1.33035362110521 | 1.33035362110521 |
